# Facilitating treatment engagement for early psychosis through peer-delivered decision support: intervention development and protocol for pilot evaluation

**DOI:** 10.1186/s40814-021-00927-8

**Published:** 2021-10-24

**Authors:** Elizabeth C. Thomas, John Suarez, Alicia Lucksted, Laura A. Siminoff, Irene Hurford, Lisa B. Dixon, Maria O’Connell, David L. Penn, Mark S. Salzer

**Affiliations:** 1 Temple University College of Public Health, 1700 N Broad Street, Philadelphia, PA 19121 USA; 2 University of Maryland School of Medicine, 737 West Lombard Street, Baltimore, MD 21201 USA; 3 Irene Hurford MD PLLC, 261 Old York Road #925, Jenkintown, PA 19046 USA; 4 Columbia University Department of Psychiatry, 1051 Riverside Drive, New York, NY 10032 USA; 5 Yale University School of Medicine, 300 George Street, New Haven, CT 06511 USA; 6 Department of Psychology & Neuroscience, University of North Carolina at Chapel Hill, 256 Davie Hall, Chapel Hill, NC 27514 USA; 7 Australian Catholic University, School of Behavioural and Health Sciences, Melbourne, VIC Australia

**Keywords:** First-episode psychosis, Young adults, Peer support, Coordinated specialty care, Shared decision making

## Abstract

**Background:**

Emerging adults with early psychosis demonstrate high rates of service disengagement from critical early intervention services. Decision support interventions and peer support have both been shown to enhance service engagement but are understudied in this population. The purposes of this article are to describe the development of a novel peer-delivered decision coaching intervention for this population and to report plans for a pilot study designed to gather preliminary data about its feasibility, acceptability, and potential impact.

**Methods:**

The intervention was developed based on formative qualitative data and in collaboration with a diverse team of researchers, key stakeholders, and expert consultants. The pilot trial will utilize a single-group (*N* = 20), pre-post, convergent mixed-methods design to explore whether and how the intervention addresses decision-making needs (the primary intervention target). The impact of the intervention on secondary outcomes (e.g., engagement in the program) will also be assessed. Additionally, through observation and feedback from the peer decision coach and study participants, we will evaluate the feasibility of research and intervention procedures, and the acceptability of information and support from the peer decision coach.

**Discussion:**

The peer-delivered decision coaching intervention holds promise for assisting young people with making informed and values-consistent decisions about their care, and potentially enhancing service engagement within this traditionally difficult-to-engage population. If the intervention demonstrates feasibility and acceptability, and pilot data show its potential for improving treatment decision-making, our work will also lay the foundation for a new evidence base regarding roles for peer specialists on early intervention teams.

**Trial registration:**

This trial was registered with ClinicalTrials.gov (Identifier: NCT04532034) on 28 August 2020 as Temple University Protocol Record 261047, Facilitating Engagement in Evidence-Based Treatment for Early Psychosis.

## Introduction

The long-term occupational, social, and economic outcomes associated with psychosis make it an urgent public health problem [1, 2]. Most clinical deterioration is expected to occur during the first 5 years after psychosis onset; this is a “critical period” during which it is essential that individuals receive appropriate treatment [3]. A shorter period between psychosis onset and receipt of appropriate care is associated with better outcomes decades later [4, 5], and moderates the effect of early treatment [6]. Coordinated specialty care (CSC) is now the gold standard in early intervention in psychosis in the USA [7], demonstrating positive clinical and functional effects in the short-term, and longer-term reduced hospitalization rates [6, 8–11]. CSC is comprehensive and team-based, often including services such as individual cognitive behavioral therapy, family education and support, supported employment and education, and evidence-based psychopharmacology for early psychosis [12].

Service disengagement among emerging adults with early psychosis (i.e., premature treatment drop-out) is a prevalent problem, with recent estimates between 20 and 40% [13]. This is particularly concerning given that service disengagement during early psychosis is a risk factor for relapse, persistent symptoms, and poorer prognosis [14]. Further, as emerging adulthood is characterized by critical developmental milestones, young people who do not receive appropriate services during this time are at increased risk for health issues and poorer functioning as they grow older [15, 16]. While some CSC programs demonstrate relatively low rates of disengagement [17, 18], many have ongoing retention difficulties, suggesting that current strategies are not sufficient to address disengagement in all contexts [19]. Especially needed are interventions that promote early and sustained engagement (i.e., help-seeking, appointment attendance, engagement in treatment processes) [20] to foster long-term retention and, thus, better outcomes.

### Sources of decisional conflict as intervention targets

Decisional conflict, a construct applicable to treatment decision-making and service engagement, is “the simultaneous opposing tendencies within the individual to accept and reject a given course of action” [21]. There are several modifiable sources of decisional conflict (hereafter referred to as “decision-making targets”) including knowledge deficiencies and a lack of social support in decision-making [22]. Given strong associations among decisional conflict, discontinuance of chosen options, and decisional regret in the general healthcare literature [23, 24], it is crucial that service engagement approaches address relevant decision-making targets. A growing literature on psychological and social barriers to engagement suggest that these decision-making targets apply to emerging adults with early psychosis [13, 25]. In our previous qualitative research, decisions about life and treatment goals, and those about psychiatric medication use were commonly described as difficult or complicated by emerging adults in early psychosis care [26]. However, there are currently no formal mechanisms facilitating swift detection of decisional conflict during CSC delivery, and decision-making strategies place a heavy emphasis on psychoeducation [12] rather than actively helping young people *use* that information to come to a decision and act on it. Information alone is not sufficient to address decisional conflict [21].

### Decision support as an intervention to promote service engagement

Decision support interventions help individuals to make evidence informed, values-consistent healthcare decisions by assessing and modifying decision-making targets. Approaches typically include decision coaching, guidance, and/or presentation of decision aids [27]. These interventions have been used to support a range of screening and treatment-related decisions for a number of physical health conditions, demonstrating improvement in decisional conflict, knowledge of health conditions and treatment options, decision quality, and acceptance of treatment recommendations [28, 29]. They have been developed for individuals who experience psychosis, often for choices about antipsychotic medication [30], but with recent application to other decisions (e.g., family involvement in care) [31]. Of import, quantitative and qualitative findings indicate that decision support interventions improve mental health service engagement [32–34]. They also facilitate shared decision-making [35], a vital part of the recovery-oriented and person-centered care that mental health service systems seek to promote [36–38], and a goal of CSC programs that emerging adults consider to be an engagement-facilitator [12, 17].

### Peer-delivered decision support to enhance decision-making and service engagement

Peer specialists, service providers with lived experience of a mental health condition [39], are in an ideal position to deliver decision support, and have the potential to significantly enhance service engagement in CSC. First, a review of the information and decision-making needs of individuals with mental illnesses demonstrated that service users value information about the lived experience of similar others; thus, peers may positively impact acceptance and uptake of decision support interventions [40]. Further, service users specifically request support from others with their own lived experience who can assist them with articulating their opinions and advocating for their rights [41]. Second, examples of peer-facilitated decision support and shared decision-making interventions in mental health exist [30, 42], supporting the feasibility of training peer specialists for this purpose. Third, studies document the effectiveness of peer-led interventions for enhancing initial and ongoing service engagement [43, 44], increasing awareness of personal challenges and the role of treatment, and promoting self-advocacy with providers [45]. This may be because peers, by virtue of their unique skills, experiential knowledge, and shared experiences, may impact decision-making targets in ways that non-peer providers cannot [20, 46–48]. Finally, there is increasing support for the peer specialist workforce—peer support is regarded as an evidence-based model of mental health care [49, 50], peer-delivered services are now eligible for Medicaid reimbursement in more than 30 states [51], and NIMH has encouraged the inclusion of peers as members of CSC teams [52]. The unique contributions of peer specialists may add to, but do not replace, what other CSC providers do to support young people with decision-making, including providing them with facts about options, answering questions, asking about personal preferences, and helping them carry out decisions.

### Objectives

Despite their promise, decision support interventions are lacking for emerging adults participating in CSC [53]. Therefore, informed by our formative qualitative data [26] and based on existing decision support materials [54], we developed a peer-delivered decision support intervention designed to improve service engagement by helping individuals make informed treatment decisions after CSC enrollment. The purposes of this article are to describe the intervention development process and report plans for a pilot and feasibility study. As such, we aim to promote transparency and accountability related to our research process, and timely dissemination of our work.

## Methods

### Intervention and development process

#### Development procedure

The investigators, a stakeholder steering committee comprised of peer specialists and a young adult CSC participant, and other consultants developed the intervention materials (i.e., manual, provider training protocols) through an iterative process. The first author was responsible for initial drafting of intervention materials, eliciting feedback from the team, and making revisions accordingly. Consistent with their areas of expertise, each investigator was responsible for ensuring that the intervention met specific requirements: (a) incorporated principles of peer support (MSS); (b) responded to results from our qualitative analysis of emerging adults’ decision-making needs (AL); (c) built upon lessons learned from development of other engagement/adherence interventions (LBD); (d) incorporated an appropriate treatment decision-making model and conformed to best practices for decision support (LAS); (e) could be feasibly implemented within the study site (IH); and (f) was appropriate for emerging adults with early psychosis (DLP). The stakeholder steering committee maximized real-world applicability of intervention content by providing feedback to ensure that it addressed the needs of emerging adults with early psychosis [55]. Finally, consultants provided input into specific aspects of the intervention according to their expertise in peer support, emerging adult decision-making, and shared decision making.

#### Theoretical model

Our conceptual approach is informed by shared decision making principles [35], and follows the evidence-based Ottawa Decision Support Framework [54] and social psychological theories underlying peer support [20, 46–48]. Shared decision-making is defined as an encounter between an healthcare provider and service user in which both are involved in a treatment-related decision, there is a bidirectional exchange of information and preferences, and there is a mutual agreement on a course of action [35]. The goal is to increase consumers’ involvement in clinical decision-making processes, which is expected to concurrently strengthen the therapeutic alliance and lead to greater satisfaction with and engagement in services [38, 56]. By clarifying individuals’ preferences and values and building communication skills to express them, the decision support intervention will prepare consumers to engage in shared decision-making with CSC providers.

The Ottawa Decision Support Framework is based on the construct of decisional conflict [21], the theory of planned behavior [57], and theories of social support [58, 59]. According to the theories that comprise the Ottawa Decision Support Framework, modifiable decision-making targets contribute to decisional conflict (Table 1). The degree to which these decision-making targets are addressed through decision support is expected to impact decision quality as well as clinical and healthcare service utilization outcomes [22]. The peer-delivered decision support intervention will address each of these decision-making targets through the various decision coaching components and peer specialist enhancements.

**Table 1:** Decision-making targets, decision coaching components, and theory-driven peer specialist enhancements

Target (A. M. O'Connor et al. 1998) [22]	Decision coaching components (Stacey et al. 2012) [56]	Theory-driven peer specialist enhancements	Theoretical Rationale (M. S. Salzer, 2002; M. S. Salzer and Kottsieper, 2015; Solomon, 2004) [20, 47, 48]
(a) Knowledge deficiencies	Provision of factual information about options, benefits, and risks; verification of understanding	Share personal experiences to enhance practical knowledge	Experiential knowledge
(b) Unrealistic expectations		Share personal experiences; provide examples of positive behaviors to correct misperceptions about mental illness and treatment	Experiential knowledge, social learning theory
(c) Unclear values	Values clarification	Encourage personal choice; explore values/preferences	Self-determination theory
(d) Social pressure	Building skills for deliberation, communication, and assessing support	Model and help consumers build communication and self-advocacy skills; provide emotional and informational support	Self-determination theory, social learning theory; social support theory; social comparison theory
(e) Lack of social support			
(f) Discrepancies between desired and actual role in decision-making			
(g) Low self-efficacy	Screening for barriers to implementation; facilitating progress in decision-making	Provide social support and model positive behaviors to enhance self-efficacy	Social support theory, self-efficacy theory
(h) Lack of resources		Provide information about resources; facilitate connection to professional and natural supports	Experiential knowledge

Theories underlying peer-delivered services [20, 47, 48] suggest unique ways in which peer specialists may enhance the decision coaching process to address decision-making targets. Experiential knowledge [60] that comes from lived experience of mental illness and recovery can provide an alternative worldview to learned knowledge obtained through non-peer providers [47]. Following social learning theory [61] and self-efficacy theory [62], behavioral observation of peers who have moved forward in recovery can combat stereotypes about people with mental health conditions, reduce internalized stigma, and enhance motivation, self-efficacy, and health-promoting behaviors [20, 47]. Peer specialists promote self-determination through their emphasis on and modeling of personal responsibility and self-advocacy [45, 63], which has implications for the sustainability of treatment-related decisions [64]. Peer-delivered services can also increase the availability of various types of social support (e.g., emotional, informational) [47], and enhance the mutuality and reciprocity of the “provider-service user” relationship compared to mainstream psychiatric practice [39], further building self-efficacy [65, 66]. Finally, peer support provides a means for social comparison [67], enhancing feelings of normalcy and connectedness.

#### Intervention elements and procedure

Decision coaching was selected as the method of decision support. Decision coaching, a process of non-directive support by a trained but neutral individual, may be used to facilitate treatment decision-making through assessment of decision-making targets and delivery of specific intervention components to address them (see Table 1) [68]. For example, these components may include facilitating access to information, clarifying values, helping a young person obtain the needed support to make a decision, and screening for implementation barriers. A trained peer specialist “decision coach” will provide the intervention. Due to having lived experience of a mental health condition, the peer decision coach will also be able to provide unique types of information and support within the coaching process (e.g., sharing personal experience to enhance practical knowledge of what it is like to choose a certain option) (Table 1).

The peer-delivered decision support intervention is comprised of three phases—relationship building, decision support, and follow-up. Grounded in Intentional Peer Support principles [69], the goals of the relationship phase (1–2 sessions) are for the peer decision coach and young person to get to know one another, build trust and connection, and jointly decide how they will work together. During the decision support phase (2–12 sessions), the peer decision coach helps the young person identify a decision that needs to be made and explores their decision-making needs. The peer decision coach utilizes a tool called the Ottawa Personal Decision Guide (OPDG), a standardized protocol shown to facilitate decision coaching [70, 71], to guide this discussion. Following identification of decision-making needs, the peer decision coach provides tailored support to address these needs by delivering relevant decision coaching components (identified in Table 1) and assists the young person with developing a decision-making plan. Finally, during the follow-up phase (1–2 sessions), the peer decision coach and young person check in at an agreed upon time to review implementation of the decision-making plan and determine whether further support is needed. Another important goal of this phase is to identify what each learned during the process of working together, and what they could do to help others through their new knowledge. The goals of each phase of the intervention are not necessarily fully achieved when the phase is completed. For example, relationship building may continue into the decision support and follow-up phases, and phases may be repeated to meet the needs and desires of the young person.

Following standards of practice for decision coaching [27], the peer specialist will deliver the intervention in face-to-face meetings or via telephone or videoconference. This flexible approach will facilitate access and engagement, and is not expected to lead to differences in outcomes given the demonstrated efficacy of both in-person and virtual decision coaching [68]. Although the intervention is designed for young adults who can make autonomous treatment decisions, individuals will be invited to involve others (e.g., family members) in conversations with the peer decision coach. Because the role of the peer decision coach is not to provide treatment advice, they are encouraged to coordinate with other CSC team members as appropriate to help the young person get the information and support they need to make decisions about their care.

The intervention recognizes decision-making as a process and not a single event [72]. The length of the decision-making process depends on factors such as how urgent the decision is, the availability of other people who may be involved in the decision (e.g. family members), and the particular decision-making needs of the young person. Therefore, the peer decision coach and young person will decide together how long the coaching process will take and how many meetings they will have. However, the duration of the intervention is expected to be between 1 and 3 months. Each decision coaching meeting is expected to be approximately 45–60 min, and it is recommended that meetings be conducted weekly or every other week. Informal check-ins may be conducted between meetings as needed.

#### Intervention materials

Based on materials to facilitate sustainable implementation of decision coaching in routine clinical practice [70], the intervention *manual* includes a discussion of difficult decision points for emerging adults, the definition and sources of decisional conflict, key Ottawa Decision Support Framework concepts, and how to use tools (e.g., OPDG) and deliver decision coaching components to facilitate decision-making. Informed by Intentional Peer Support principles [69], the manual also stresses the importance of mutuality and co-learning processes and provides guidance related to using positive self-disclosure to benefit peers. A *peer specialist training protocol* consists of mandated human subjects’ protection education and discussion of the procedure for addressing adverse events, reviewing the intervention materials, and learning about the concept of decisional conflict. Incorporating training-related recommendations by the Ottawa Decision Support Framework group [70], activities include completion of an online Ottawa Decision Support Tutorial (https://decisionaid.ohri.ca/odst/odst.php) that provides an introduction to decision support concepts, discussion of how these concepts apply to supporting young adults with decision-making, and role plays to allow practice of components of decision coaching. A *CSC staff training protocol* consists of learning about emerging adults’ treatment decision-making needs, characteristics of the peer decision coaching intervention, and study eligibility criteria and procedures. Staff also learn about how to facilitate implementation of the intervention.

### Pilot study protocol

#### Overall strategy

The pilot study will utilize a single-group, pre-post, convergent mixed methods design to explore whether and how the intervention engages decision-making targets. This design will enable us to link themes regarding individuals’ experience of the intervention with a quantitative measure of decision-making targets [73]. We will also evaluate the feasibility of research and intervention procedures, and the acceptability of information and support from the peer specialist.

#### Study setting

Participants will be recruited from two CSC program sites in the northeastern USA. The CSC program sites consist of multidisciplinary teams, including peer specialists who will serve as study interventionists, and offer community outreach/engagement, rapid access to care, comprehensive assessment, collaborative treatment planning and shared decision-making, peer support, recovery-oriented cognitive behavioral therapy, care management and coordination, family education and support, occupational therapy and supported employment/education, and evidence-based psychopharmacology for early psychosis.

#### Eligibility criteria

The inclusion criteria are (1) 18–30 years of age; 2) experiencing early psychosis, defined as psychosis lasting 18 months or less between the time when threshold symptom criteria were reached (as determined by the admitting CSC program assessor) and the date of CSC program enrollment; (3) able to speak/understand English; (4) able to provide informed consent as assessed by research staff; and (5) enrolled in the CSC program for any period of time. The *exclusion criteria* are: having a legal guardian or diagnosis of dementia, delirium, or intellectual disability as determined by the admitting CSC program psychiatrist.

#### Outcomes

The primary outcomes associated with this study pertain to feasibility, acceptability, and decision-making targets, as assessed by quantitative and qualitative measures.

#### Feasibility

We will track data pertaining to recruitment, retention, and assessment procedures following CONSORT guidelines [74].

We will utilize a multi-pronged approach to assess intervention fidelity and implementation. First, participants will be asked to provide permission to audio record intervention meetings so that fidelity may be assessed. Participants may opt out of audio recording of intervention sessions and will still be able to participate in the intervention. The first author and a trained research assistant will randomly select and complete the Decision Support Analysis Tool (DSAT), a reliable and valid evaluation tool to assess health practitioners’ decision support and communication skills [75], for 10% of audio-recorded meetings, stratified by participant. Second, the peer decision coach will complete a contact note after each meeting documenting meeting length and mode (i.e., telephone, videoconference), components of the intervention that were covered, and any reasons for deviations from the manual. Third, we will collect information from participants about the perceived presence of intervention elements during contacts with the peer decision coach via the Qualitative Measure of Target Engagement (described below) and the Intentional Peer Support Core Competencies Scale [76], a measure designed to assess the relationship between a peer specialist and service user.

#### Acceptability

Following recommendations by the International Patient Decision Aid Standards (IPDAS) group [77], we developed a short survey to assess participants’ perceptions about the amount, clarity, helpfulness, and balance of the information provided by the peer decision coach. We also included open-ended, qualitative questions to gather information about individuals’ experience of the intervention, its perceived strengths and weaknesses, and the value of having a person with lived experience of a mental health condition provide decision support. Examples of open-ended questions include: “Overall, what did you think about the decision support you received from the peer specialist?” “What did you like most?” “What did you like least?” “Was it helpful having someone to talk to who had experience of a mental health condition? If ‘yes,’ why was it helpful? If ‘no,’ why not?”

#### Quantitative measure of decision-making targets

The Decisional Conflict Scale (DCS) [78] is the most commonly used assessment in research on decision support [79]. It contains 16 items, rated on a Likert scale (0 = strongly agree, 4 = strongly disagree), and three subscales: decision uncertainty, factors contributing to uncertainty, and perceived effective decision-making. It has adequate reliability (α = .78–.92; test-retest *r* = .81), and discriminates between those who accept treatment, reject treatment, and delay decisions [78]. The factors contributing to uncertainty subscale measures decision-making targets (e.g., knowledge, values, support, self-efficacy). We will use scores from this subscale in the planned analyses.

#### Qualitative measure of decision-making targets

To gather information about the degree to which participants feel that the peer decision coach addressed each decision-making target and the perceived impact of the information and support received, we developed a qualitative interview of decision-making targets. Questions parallel DCS items (e.g., “Did the peer decision coach talk with you about options associated with your decision? If yes: Please describe how s/he did this. Did this impact your decision? Why or why not? If no: Would this have been helpful to you? Why or why not?”). Data from this assessment will foster a greater understanding of how various types of information and support received from the peer decision coach contributed to decision-making.

### Other measures and exploratory outcomes

#### Demographic and clinical characteristics

Demographic and clinical characteristic data will be obtained by participant self-report. Demographic characteristics will include age, sex, race/ethnicity, education level, employment status, and forensic history. Clinical characteristics will include psychiatric diagnosis, current use of substances, family involvement in treatment, and psychotropic medications.

A number of exploratory outcomes will also be assessed using the following measures:

#### Multidimensional Scale of Perceived Social Support [80]

This scale assesses perceived adequacy of support in the following areas: family, friends, and significant other. It has been shown to demonstrate good internal consistency and test-retest reliability and moderate construct validity [80]. Total scores will be used in the planned analyses.

#### Control Preference Scale [81]

This measure assesses patients’ preferences for participation in treatment decision-making. The scale has been tested in a range of populations and has proven to be reliable and valid [81]. Total scores will be used in the planned analyses.

#### Perceived Involvement in Care Scale [82]

This scale measures perceived clinician facilitation of service user involvement in decision-making, perceived level of information exchange between service user and provider, and perceived level of the service user’s own involvement in medical decision-making. Internal consistency has been shown to be acceptable, and the measure demonstrates content validity [82]. Subscale and total scores will be used in the planned analyses.

#### Birchwood Insight Scale [83]

This scale measures dimensions of insight in the following areas: ability to re-label symptoms, awareness of mental illness, and recognition of a need for treatment. The scale demonstrates evidence of reliability, validity, and sensitivity to change [83]. Total scores will be used in the planned analyses.

#### Recovery Assessment Scale [84]

This scale measures personal recovery and consists of 5 factors: personal confidence and hope, willingness to ask for help, goal and success orientation, reliance on others, and not being dominated by symptoms. The Recovery Assessment Scale shows good internal consistency, test-retest reliability, and interrater reliability [85]. Total scores will be used in the planned analyses.

#### Internalized Stigma of Mental Illness Scale [86]

This measure is designed to assess individuals’ experience of stigma related to mental illness. It has been shown to have good internal consistency, test-retest reliability, and construct validity [86]. Total scores will be used in the planned analyses.

#### Empowerment Scale [87]

This scale measures empowerment, control, self-determination, and decision making in the recovery process. The Empowerment Scale shows a high degree of internal consistency and evidence of validity [87]. Total scores will be used in the planned analyses.

#### Decision-Self-Efficacy Scale [88]

This measure assesses confidence in making an informed treatment choice. The scale demonstrates adequate internal consistency and discriminant validity [88]. Total scores will be used in the planned analyses.

#### Service Use and Resources Form—Monthly [89]

This is a self-report measure that assesses service use over the past month from diverse sources of inpatient and outpatient care including antipsychotic medication with daily doses, and other psychotropic and non-psychotropic medication. It has been utilized in clinical trials of both pharmacological and psychosocial interventions for people with serious mental illnesses [90, 91]. Number of outpatient mental health visits, derived from the SURF, will be used in the planned analyses.

#### Brief Adherence Rating Scale [92]

This scale assesses antipsychotic medication adherence of service users in outpatient settings. It has been shown to be valid, as indicated by a strong relationship with adherence as assessed by electronic monitoring; the scale also demonstrates acceptable internal consistency and test-retest reliability [92]. Total scores will be used in the planned analyses.

#### Intent to Attend Measure [6]

This is a two-item measure utilized during a large clinical trial of early intervention services in the USA that assesses participants’ intentions to attend their next treatment appointment and to participate in treatment at a CSC program for the recommended 2-year period [6].

#### Service Engagement Scale [93]

This is a clinician-rated scale that measures service users’ engagement in community mental health services. The Service Engagement Scale shows good face validity and content validity, high test-retest reliability, and internal consistency [93]. Total scores will be used in the planned analyses.

#### Working Alliance Inventory [94]

This 36-item scale assesses the strength of the therapeutic alliance with participants’ therapists in three domains: agreement on goals, agreement on tasks, and the development of bonds. It has adequate reliability, and demonstrates convergent, discriminant, concurrent, and predictive validity [94]. Total scores will be used in the planned analyses.

#### Satisfaction with Decision Scale [95]

This scale assesses a patient’s satisfaction with a health care decision. It has excellent internal consistency and good convergent and discriminant validity [95]. Total scores will be used in the planned analyses.

#### Procedures and timeline

During the month prior to beginning participant recruitment and enrollment, the first author will train the peer specialists and CSC staff at the study sites using the peer specialist and CSC staff training protocols. The peer specialist training will be conducted in 3 6-hour sessions. Peers will also complete individual exercises between training sessions, such as completing online human subjects’ protection education. The CSC staff training will be conducted in 1 1-hour session.

As shown in Table 2, participants will complete a baseline assessment with the research assistant prior to engaging in the peer-delivered decision support intervention.

**Table 2:** Measures and time points

	Baseline	Post-intervention
Demographics/clinical characteristics	X	
Multidimensional Scale of Perceived Social Support	X	X
Control Preference Scale	X	X
Perceived Involvement in Care Scale	X	X
Birchwood Insight Scale	X	X
Recovery Assessment Scale	X	X
Internalized Stigma of Mental Illness Scale	X	X
Empowerment Scale	X	X
Decision Self-Efficacy Scale	X	X
Service Use and Resource Form—Monthly	X	X
Brief Adherence Rating Scale	X	X
Intent to Attend Measure	X	X
Service Engagement Scale	X	X
Quantitative Measure of Decision-Making Targets (Decisional Conflict Scale)	X	X
Working Alliance Inventory	X	X
Satisfaction with Decision Scale		X
Qualitative Measure of Decision-Making Targets		X
Acceptability Measure		X

Following the baseline assessment, participants will engage in the experimental peer-delivered decision support intervention and will maintain access to other services and supports normally available to them through their respective CSC programs (e.g., medication, therapy). During weekly supervision with the first author, the peer decision coach will review contacts with individuals, and discuss logistical, ethical, and fidelity issues. During regular team meetings, the peer decision coach will also be in communication with other CSC providers to support participants’ decision-making as appropriate.

Upon completion of the intervention, participants will complete a post-intervention assessment with the research assistant. At post-intervention, participants will be asked to provide permission to audio-record responses to qualitative questions that are part of the qualitative assessment of decision-making needs and acceptability measure. Participants must provide permission to audio-record these portions of the research interview in order to participate in the study. At baseline and post-intervention, the study team will reach out to participants’ psychiatrists/therapists in order to have them complete the Service Engagement Scale. Participants will be compensated $20 in gift cards for completing the pre-intervention assessment, and $30 in gift cards for completing the post-intervention assessment.

#### Sample size

We will recruit and collect data from 20 individuals enrolled in the CSC programs who will participate in the intervention. Given that this study’s purpose is to collect preliminary data about the intervention’s engagement of targets, the sample size is based on pragmatics rather than power, consistent with recommendations for pilot studies [96]. We used the current rate of the CSC program enrollment and a conservative estimate of study enrollment of 40% to arrive at the projected N.

#### Recruitment

Recruitment strategies that will be utilized to enroll participants in the study include distributing flyers and completing site presentations. Additionally, we will collaborate with CSC staff members who will provide information about the study to potentially eligible individuals, and the research assistant will follow up with those interested in participation for screening.

#### Data management

Completed interviews will contain a coded identification number to prevent loss of confidentiality, and any identifying information will be removed from interview transcripts. Confidentiality of data files will be achieved by separating code numbers from individual identifying information. Information taken about participants will only be kept electronically in encrypted, password protected files and hard copies will be stored in locked cabinets in a locked office. Data sources containing identifiers (i.e., regulatory documents such as the eligibility screener and contact information form, clinical characteristic form) will always be kept separate from other research data in encrypted, password-protected files. Participant files will only be made available to personnel involved in the study through the use of access privileges and passwords. All published reports will contain data reported either in aggregate form (where no individual responses can be identified), or in composite individual examples that are constructed so that identification is impossible. Individual examples or quotations that may be presented in published reports will use pseudonyms, so that participants’ identity will be protected. Audio recordings will be secure and confidential per the transcription service provider’s non-disclosure agreement and encryption software. Audio-recordings will be immediately deleted from the recorder after successful uploading to the transcription provider’s secure site. All other data collected from this study will be kept for 7 years after the last publication.

#### Statistical methods

Demographic and clinical variables and feasibility data will be reported using descriptive statistics.

Quantitative acceptability data will be reported using descriptive statistics. The first author and a trained research assistant will analyze responses to open-ended questions using the Constant Comparison Method [97]. As open-ended questions do not parallel survey questions, acceptability data will be interpreted and reported contiguously [73].

To examine within-group differences in quantitative decision-making targets pre- and post-intervention, we will conduct a paired samples *t* test using DCS Factors Contributing to Uncertainty subscale scores. Responses to open-ended questions about decision-making targets will be analyzed qualitatively using the same procedure described previously. The parallel structure of the qualitative interview to the DCS will enable quantitative and qualitative data to be merged and reported through narrative weaving [73]. Integration of these data will provide a more nuanced understanding of whether and how the intervention engages decision-making targets than use of either type of data alone, and will be most informative for intervention refinement. Should quantitative and qualitative analyses yield discrepant findings, we will assess reasons for conflicting results (e.g., low power, question structure/content) and revise procedures accordingly [73].

Paired samples *t* tests will also be used to examine pre-post changes in exploratory outcomes. We will calculate and assess effect sizes for all *t* tests using Cohen’s *d* [98]. Throughout, 95% confidence intervals will be presented.

### Monitoring

#### Data and safety monitoring committee

A Data and Safety Monitoring Committee, comprised of researchers and people with lived experience of a mental health condition and independent from the sponsor and competing interests, will meet at least annually to perform monitoring functions and activities, and to review regulatory documentation. Additional meetings will be scheduled as needed, or in the case of adverse events. The Committee will oversee interim analyses, where appropriate, to determine whether protocols should be modified or terminated either to minimize risks for subjects or to conserve research resources in cases where the risks versus benefits of the study are apparent early, or when early data indicate that the current design is unlikely to be informative. The Committee will recommend terminating a study due to unacceptable changes to the knowledge regarding the potential risks associated with participation.

#### Harms

The research assistant will routinely administer an adverse events checklist at each study visit, and the peer decision coach will monitor for adverse events during each intervention meeting. If an adverse event occurs during a participant’s participation in the study, the peer decision coach or research assistant will facilitate participants’ access to prompt medical or professional care as appropriate, document the event on an adverse event reporting log, and immediately submit documentation of the event to the IRB and NIMH as appropriate.

#### Auditing

In addition to annual review by the Data and Safety Monitoring Committee, this project will be subject to routine audits within the Temple University and Philadelphia Institutional Review Boards (IRB). Annual status reports will be made to the National Institute of Mental Health.

### Ethics and dissemination

#### Confidentiality

Protected health information (PHI) collected during the study is limited to participant’s self-reported contact information (e.g., name, address) and psychiatric diagnosis. PHI will not be disclosed, except as may be required by law. Any information about child abuse or intent to harm self or others will be reported to authorities, as required by law. Research staff will be the only people with access to this data, with the exception of authorized representatives of the Temple University or Philadelphia Institutional Review Board (IRB), the National Institute of Mental Health (the study sponsor), and the Office of Human Research Protections (OHRP).

#### Ancillary and post-trial care

In the event that a participant has been harmed by the research, we will facilitate prompt receipt of their psychiatric or medical care. However, participants will be responsible for the costs of such psychiatric or medical treatment, directly or through medical insurance and/or other forms of medical coverage. Temple University and research staff will not be responsible for the cost of this treatment. This has been made explicit on the informed consent forms. Participants will maintain access to services normally offered at their respective CSC programs during and after the trial.

#### Dissemination policy

In accordance with study sponsor guidelines, this study will be registered and de-identified results information (including participant flow, demographic and baseline characteristics, outcomes and statistical analyses, adverse events, the protocol and statistical analysis plan, and administrative information) will be submitted to ClinicalTrials.gov.

## Discussion

This pilot and feasibility study is subject to limitations common to other pilot trials. Sample size is small, limiting the ability to detect changes in study outcomes. Further, as it is not a randomized controlled trial, any changes in outcomes that are observed cannot be definitively attributed to participation in the intervention. However, demonstration of intervention efficacy is beyond the scope of this pilot trial and the purpose of administration of outcome measures is to assess their feasibility and acceptability in preparation for a larger scale study. In addition, the mixed methods approach will enable us to gather preliminary data about whether and how the intervention may impact outcomes. Further, as the study is only being conducted at two sites, it may not be generalizable to other CSC programs. Study results will be used to inform intervention refinement so that it may be scaled up and implemented across a larger number of CSCs.

To our knowledge, the peer-delivered decision support intervention described herein will be the first of its kind to assist emerging adults with making treatment-related decisions in the context of CSC. Decision support for this purpose holds promise for enhancing service engagement. Further, if the intervention demonstrates feasibility and acceptability and pilot data show its potential for improving treatment decision-making, our work will lay the foundation for a new evidence base regarding roles for peer specialists on early intervention teams. With increasing attention to including peer specialists on early intervention teams but little guidance as to what their roles should be [52, 99], this project is particularly timely.

## Data Availability

Not applicable

## Abbreviations

CSC: Coordinated specialty care
CONSORT: Consolidated Standards of Reporting Trials
DCS: Decision Conflict Scale
DSAT: Decision Support Analysis Tool
IPDAS: International Patient Decision Aid Standards
IRB: Institutional Review Board
NIMH: National Institute of Mental Health
OHRP: Office of Human Research Protections
OPDG: Ottawa Personal Decision Guide
PHI: Protected Health Information
RAISE: Recovery After an Initial Schizophrenia Episode
